# Screening for Infectious Diseases among Newly Arrived Migrants: Experiences and Practices in Non-EU Countries of the Mediterranean Basin and Black Sea

**DOI:** 10.3390/ijerph121215002

**Published:** 2015-12-08

**Authors:** Christian Napoli, Maria Grazia Dente, Tommi Kärki, Flavia Riccardo, Pasqualino Rossi, Silvia Declich, the Network for the Control of Cross-Border Health Threats in the Mediterranean Basin and Black Sea

**Affiliations:** 1 National Centre for Epidemiology, Surveillance and Health Promotion, National Institute of Health (Istituto Superiore di Sanità, ISS), viale Regina Elena 299, Rome 00161, Italy; mariagrazia.dente@iss.it (M.G.D.); tommi.karki@iss.it (T.K.); flavia.riccardo@iss.it (F.R.); silvia.declich@iss.it (S.D.); 2 European Programme for Intervention Epidemiology Training (EPIET), European Centre for Disease Prevention and Control (ECDC), Tomtebodavägen 11a, Stockholm 17183, Sweden; 3 DG Communication and European and International Relations—Italian Ministry of Health, Lungotevere Ripa 1, Rome 00153, Italy; p.rossi@sanita.it; 4 Network for the Control of Cross-border Health Threats in the Mediterranean Basin and Black Sea—Coordination Centre, National Institute of Health (Istituto Superiore di Sanità, ISS), viale Regina Elena 299, Rome 00161, Italy; episouth@iss.it

**Keywords:** screening for infectious diseases, newly arrived migrants, Mediterranean Basin and Black Sea

## Abstract

Changing migration dynamics in the Mediterranean Sea and differences in infectious diseases (ID) burden between the countries of origin have raised questions whether public health actions are needed to avoid the transmission of ID. Screening newly arrived migrants for ID is one health monitoring option, offering opportunities for prevention, early detection and treatment. The authors conducted a survey among country experts in non-European Union countries of the Mediterranean and Black Sea, in order to explore current ID screening practices and policies for newly arrived migrants. The association between the existence of guidelines and the proportion of refugees in the population was also estimated. Eighteen country experts responded (90%) out of the 20 invited. Eleven countries (61%) implemented screening programmes and six (38%) had national guidelines. Screening was performed most often for tuberculosis at the holding level. A higher proportion of refugees in the population was associated with the existence of guidelines for screening (*p =* 0.05). Fourteen experts (88%) considered screening among migrants useful. The results show that screening for ID in newly arrived migrants is relevant for non-EU countries of the Mediterranean and Black Sea. Common guidelines could be promoted focusing on both individual and public health benefits of screening programmes.

## 1. Introduction

The Mediterranean Sea has seen population movement since the beginning of civilization due to conflicts, commerce and pilgrimage. More recently, tourism and economic migration have also emerged as additional drivers of population movement. The traditional regional migration flows have recently acquired a more global significance, as northern African and Balkan Countries have become major transit routes for different migrant populations into Europe [1]. The European Agency for the Management of Operational Cooperation at the External Borders of the Member States of the European Union (Frontex) identifies three main migration routes across the Mediterranean basin towards the European Union: the Western Mediterranean route with sea passage from North Africa to the Iberian Peninsula; the Central Mediterranean route from Northern Africa towards Italy and Malta; and the Apulia and Calabria (Southern Italy) route, both with departure from Turkey and Egypt across the Aegean Sea and secondary movements from Greece to Italy [2]. Since 2011, the Central Mediterranean migration route has been increasingly used as an irregular entryway to the European Union (EU) in extremely dangerous circumstances. In 2014, Frontex detections of irregular migrants in the Central Mediterranean area reached unprecedented numbers with over 170,000 migrants reaching Italy (the largest influx registered into a single EU country). Additionally to these routes, three land migratory routes have also been described across the eastern borders of the EU: the Eastern Mediterranean route, the Western Balkan route and the Eastern Borders route (involving several Black Sea countries) [2].

Changing migration dynamics and routes, increased migration flows and known differences in communicable diseases burden between the countries of origin and destination of migrants have led health authorities to question whether public health actions should be considered to avoid the transmission of infectious diseases (ID) [3,4]. Screening newly arrived migrants for selected ID could be an option to consider both in the transit countries as well as in the countries of the final destination of migrant populations, as screening could improve health status monitoring, identify new symptomatic and asymptomatic cases of infection and offer opportunities for prevention, early detection and treatment [5,6,7].

In 2014, a survey on screening practices among newly arrived migrants was conducted among 27 EU/European Economic Area (EEA) countries and Switzerland in order to establish if and how these countries implemented screening programmes targeting newly arrived migrants [8]. However, no data exists on what screening practices are being implemented in countries bordering the Southern Mediterranean and Black Sea, nor how their national policies are influenced by the migration flows. In order to address this information gap and promote sharing information among countries bordering the EU, we conducted a study to map the current ID screening practices and policies for newly arrived migrants in non-EU countries of the Mediterranean and Black Sea regions.

## 2. Materials and Methods

We used a 15-point questionnaire on screening among newly arrived migrants in EU/EEA and Switzerland for the non-EU countries of Mediterranean and Black Sea regions [8]. The survey addressed in November 2014, a web-based questionnaire to focal points (FP) of the “*Network for the Control of Cross-border Health Threats in the Mediterranean Basin and Black Sea*”, appointed by the governments of 20 non-EU Member States (Albania, Algeria, Bosnia and Herzegovina, Egypt, former Yugoslav Republic of Macedonia/FYROM, Israel, Jordan, Kosovo, Lebanon, Libya, Morocco, Montenegro, Palestine, Serbia, Tunisia, Turkey, Georgia, Armenia, Moldova and Ukraine). The Network, established with the EpiSouth and the EpiSouth Plus Projects [9] and previously involved in other migrant health studies [10] has further developed with the involvement of countries bordering the Black Sea. The FP were experts in communicable diseases from relevant Ministries and National Health Institutions. Each FP coordinated the collection of data for the questionnaire in their country, also involving other national experts when appropriate. Those who did not reply to the questionnaire after the initial contact, were reminded by e-mail or by phone.

We defined screening as a systematic practice of medical examination, involving laboratory and/or other diagnostic testing, for searching and identifying cases of a specific ID in a target population. We defined newly arrived migrants by adapting the United Nations definition of migrants as persons, other than travellers or tourists, who had arrived in the previous year (less than 12 months) to a country other than their usual residence [11].

We investigated the current screening practices and the presence of guidelines in each country, both at national and subnational level. For each implemented screening programme, we asked respondents to specify which diseases were screened for, at what level in the migration process screening took place, what was the target population and whether the screening was compulsory. The different levels for screening were defined: (I) pre-entry level, as screening before entering or travelling to the receiving country; (II) entry level, as screening at the point of entry (e.g., ports or airports); (III) holding level, as screening in the migrant centres defined as reception/holding/transit facilities; (IV) community level, as screening after arrival and after partial integration to the community in the receiving country (e.g., in the primary care). Potential target populations for screening were defined as: (I) all newly arrived migrants; (II) asylum-seekers; (III) arrivals from endemic areas; (IV) other target groups, with a possibility to further specify. More than one level and target population could be indicated.

We also asked respondents to describe whether the screening data collected from their implemented programmes were generally available for public health purposes, and what actions, such as vaccination campaigns, treatment or control measures were taken based on the screening results. Finally, we asked respondents for their expert opinion on the general usefulness of screening programmes targeting newly arrived migrants. We performed a frequency analysis for all categorical variables. As not all invited countries answered the survey and not all those who answered replied to all the questions, we present the results both as crude numbers and as proportions of responses calculated on the basis of the number of respondents for each question. In order to perform an additional analysis, we acquired data on refugees (as a proxy for newly arrived migrants) and resident populations from the Department of Economic and Social Affairs of the United Nations(UN-DESA) for the year 2013 [11]. UN-DESA defines refugees as: “*Persons who are recognized as refugees under the 1951 Convention relating to the Status of Refugees and its 1967 Protocol or under the 1969 Organization of African Unity Convention Governing the Specific Aspects of Refugee Problems in Africa*; *those granted refugee status in accordance with the United Nations High Commissioner for Refugees (UNHCR) Statute*; *those granted humanitarian status or temporary protection by the State in which they find themselves*; *those in refugee-like situations*; *and Palestinian refugees registered with UNRWA*” [11]. Only those Countries for which UN-DESA provide data were considered. Based on this data we ranked and categorized the countries into three groups on the basis of the proportion of refugees in the population: low (<25/100,000), medium (25–400/100,000) and high (>400/100,000). We studied the association between this parameter and the implementation of screening programs and national guidelines by using a Fisher’s exact test. Data were analyzed by using STATA version 11.0 (StataCorp LP; College Station, TX, USA; 2009).

## 3. Results

Of the 20 country experts enrolled, 18 (90%) submitted a valid completed questionnaire. All but one of the respondents were experts from National Institutes of Public Health or from National Ministries of Health, one respondent being from Ministry of Civil Affairs.

Fifty percent of country experts (8/16) indicated that, in their opinion, migrants are having an impact on infectious disease epidemiology in their country (Table 1).

**Table 1:** Answer to the Yes/No/Don’t know*-*questions of the survey.

Question	Answer			No Answer
	Yes	No	Don’t Know	
Would you say that newly arriving migrants are having an impact on infectious disease epidemiology in your country?	8	5	3	2
Does your country routinely screen newly arriving migrants for infectious diseases on national or subnational level?	11	6	1	0
Does your country have national guidelines for screening of infectious diseases among newly arriving migrants?	6	9	1	2
Does your country routinely use migration centres for administrative detention of asylum seekers and irregular migrants?	9	4	3	2

As shown in Table 1, screening among newly arrived migrants was implemented in 61% (11/18) of responding countries, more frequently at a national level (10 countries, of which five were screening both at the national and subnational levels). One country reported only regional/subnational screening. National guidelines for screening among newly arrived migrants, at least for one disease, were available in 38% (6/16) of responding countries. In all the countries where guidelines were available screening was implemented. In five countries screening was implemented without national guidelines or reference documents.

Guidelines for screening among newly arrived migrants were more often present in countries with a high or medium proportion of refugees compared to countries with a low proportion of refugees (*p* = 0.05) (Table 2). Although countries with a higher proportion of refugees were also found to implement screening programmes more frequently compared with other countries, this difference was not statistically significant(*p* = 0.53). It should be noted that this analysis excludes two countries: one for which the respondent replied “I don’t know” and one for which UN-DESA data is not available.

**Table 2:** Association between the proportion of refugees in the population in 2013 and the implementation of screening programs and guidelines for screening.

Groups of Countries	Number of Respondents *	Proportion of Refugee in the Population			*p*-Value **
		Low Proportion	Medium Proportion	High Proportion	
Countries with guidelines for screening	14	25% (1/4)	0% (0/4)	83% (5/6)	0.05
Countries with implemented screening programs ***	15	50% (2/4)	50% (3/6)	83% (5/6)	0.53

* Number of respondents who answered “Yes” or “No” to the questions. Respondents who answered “I do not know” were not considered in this analysis; ** Fisher’s exact test; *** Analysis performed using data of countries for whom UN-DESA migrant data was available (one Country is not included).

**Figure 1:**
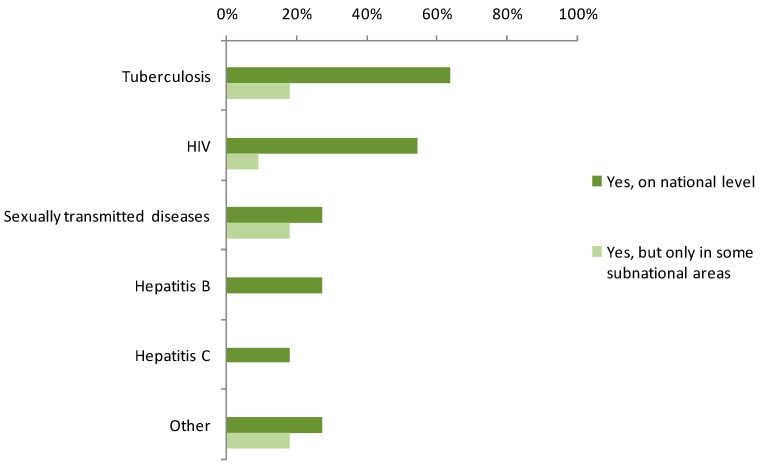
Infectious diseases screened for (number of responding countries, *n* = 11).

Most respondents (9/11; 81%) from countries implementing routine screening programmes, reported screening for tuberculosis (TB) which was, therefore, the most frequently screened ID, mainly at national level (Figure 1). Other diseases screened for included HIV (7/11; 64%), hepatitis B (3/11; 27%), sexually transmitted diseases (STD) (3/11; 27%) and hepatitis C (2/11; 18%). Forty-six percent of the experts reported screening activities in their country for other diseases/etiological agents (e.g., *Salmonella typhi*, other species of *Salmonella* spp., malaria, leprosy).

Vaccination status was checked as part of screening practices in 90% (10/11) of countries implementing screening among newly arrived migrants, almost always at national level. In one case TB vaccination was checked only at subnational level. At national level, vaccination status was checked mainly for poliomyelitis (5/10; 50%), measles (4/10; 40%), TB (3/10; 30%), hepatitis B (3/10; 30%), mumps (3/10; 30%), rubella (3/10; 30%), diphtheria (3/10; 30%) and other (2/10; 20%) (*Haemophilus influenza b*, tetanus, pertussis).

**Figure 2:**
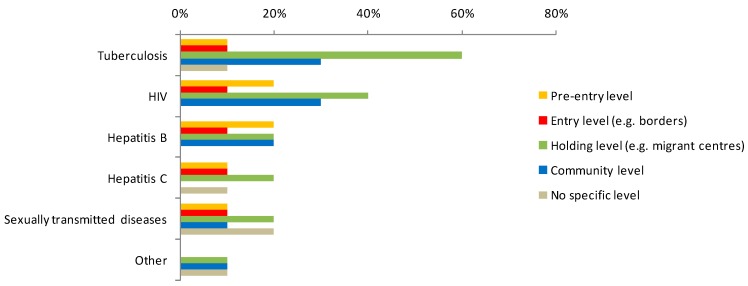
Level of screening among newly arrived migrants by disease (*n* = 10).

As shown in Table 1, migration centres are routinely used for the administrative detention of asylum seekers and irregular migrants in 56% of countries (9/16). Screening was most often performed at the holding level, particularly for TB (60% of respondents reported screening for TB at holding level and 30% at the community level). Community level screening and pre-entry screening were reported by a minority of countries (Figure 2). Screening for HIV took place at different levels in different countries (40% of respondents reported HIV screening at holding level, 30% at community level, 20% reported pre-entry and 10% entry level screening). Hepatitis B screening was performed at pre-entry, holding and community level by two countries, respectively, and at entry level by one country.

The target groups included all newly arrived migrants (36%); those from selectedcountries of origin (endemic areas) (36%); those with selected migrant-status (27% indicated screening only targeted asylum-seekers). Other target sub-groups (mainly migrant workers) were reported by 27% of the responding countries. Screening was always reported as compulsory (11/11).

The data on screening were collected and available for public health purposes in 73% (8/11) of the countries. The screening was reportedly performed in order to guide individual/public health action, including isolation or other control measures (82%), treatment in case of disease detection (82%), information on possible public health threats to international health authorities (73%), vaccination campaigns (73%), improvement of access to the national health care system (64%), pre/post-screening counselling (64%), and other activities (27%).

**Figure 3:**
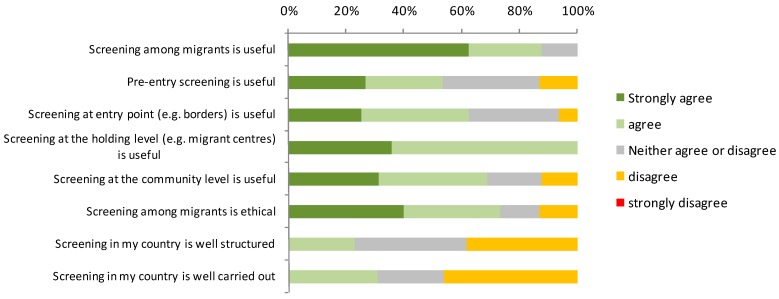
General opinions on screening among migrants (*n* = 16 countries both performing and not performing screening).

With regard to the collected expert opinions half of all respondents (8/16) reported that newly arrived migrants were impacting on the epidemiology of ID in their country. Screening among migrants was considered useful (agree or strongly agree) by 88% (14/16) of respondents, especially if the screening was conducted at holding level (14/16; 88%) (Figure 3). Community level screening was considered useful by 69% (11/16), entry level screening by 63% (10/16) and pre-entry screening by 50% (8/16) of respondents. Screening was assessed as well-structured and well carried out, by three and four countries, respectively.

When considering only those countries that perform screening, the percentage of experts believing that screening is useful was still high but decreased at 82%, and screening performed in their own countries was not often (4/11; 36%) considered well carried out and well-structured.

## 4. Discussion

Scientific evidence has shown that screening can be reasonably cost-effective and useful in helping to reduce the burden of disease for infections such as TB or hepatitis B. However how, where and who to target to improve effectiveness remains debated [12,13,14,15]. Migrant screening for ID is a topic in which this debate is particularly lively due to the lack of strong evidence regarding cost-effectiveness in this population, lack of proof of substantial transmission of diseases, like TB, from migrants to host populations that can be prevented by screening and concurrent issues related to equity and ethics [16]. Moreover, although some studies advocate for post-entry screening at holding level as the most appropriate site of intervention [17], not all newly arrived migrants can be reached in holding sites and some ID might be detected only years after the migration. In this second scenario, migrants are more likely to be dispersed in the community and might experience a more advanced disease stage. Therefore, for some ID, community level screening, in order to provide the needed treatment as soon as possible, might also be appropriate. Community level screening might also facilitate migrants’ access to follow-up health care services, that is known to be hindered by formal and informal barriers that depend on the migration status of individuals and the host country legislation [10,18].

In the case of chronic infectious diseases such as TB, hepatitis B and C, screening has, in fact, been advocated as a tool to permit an early diagnosis for treatment and secondary/tertiary prevention among higher risk migrant or otherwise vulnerable populations [17,19]. Moreover, based on the available very low quality evidence, WHO issued conditional recommendations on active TB screening for subpopulations that have very high TB rates or very poor access to health care, stressing the need of prioritizing only groups with very high risk [17]. From the perspective of low incidence countries, where the proportion of TB burden is usually highest in foreign-born populations, this recommendation might apply to migrants, regardless of status, from high prevalence countries.

In this debated context, our study highlighted that almost two thirds of the country experts we interviewed reported currently implementing national and/or subnational screening programs targeting newly arrived migrants. This is coherent with a generalized perception that migration is impacting ID epidemiology at national level in countries of the Mediterranean and Black Sea region. These expert opinions indicated that implementing ID screening programmes targeting newly arrived migrants might be useful in the Mediterranean and Black Sea region. In our study we highlighted a variety of actions being taken by countries on the basis of screening results, e.g., in a majority of countries screening is followed by treatment programmes and international health regulations procedures. This confirms previous findings showing that screening programmes and their results can provide useful information to guide actions both for the benefit of the individual and of public health and are thus valuable tool for monitoring infectious diseases among migrants [8].

Notwithstanding, most of the experts also indicated that screening performed in their own countries is not always well carried out and/or well structured. Vaccination status was reported to be checked in the context of screening activities however, with the exception of poliomyelitis, this occurred in less than half of the countries. National guidelines are also not widely available. These findings suggest that there may be areas for improvement of screening programmes targeting newly arrived migrants in this region and that there may be a need for further guidance on this topic.

Current policies regarding screening of infectious diseases among newly arrived migrants show some similarities between countries of the Mediterranean and Black Sea Region and members of the EU/EEA. Almost the same percentage of countries reported performing ID screening targeting newly arrived migrants in the two regions (59% EU/EEA *vs.* 61% Non-EU) and considered screening useful (96% EU *vs.* 88% Non-EU) [8].

Consistently with previous international studies [6,7], TB screening was the most frequently implemented programme in countries of the Mediterranean and Black Sea. Screening programmes however also targeted other diseases such as HIV and STDs, in the majority of these countries, although implementation varied. This denotes a difference with prevalent practices found in EU countries where TB is the main focus of attention of screening programmes [8].

In the Mediterranean and Black Sea countries when screening was implemented, it was post-entry, took place mostly at holding level and was always reportedly compulsory. Target groups for screening often included all newly arrived migrants, regardless of status, or migrants from endemic countries for the screened ID. In the EU/EEA, screening also mainly takes place at holding level, however compulsory screening is less frequent and asylum-seekers are the most commonly targeted migrant group [8].

Looking at the results of the analysis taking into account the proportion of refugees or asylum-seekers in the population, in both EU and non-EU countries the proportion of recent migrant groups in the population was associated with the existence of guidelines for screening [8]. However, the implementation of screening and the proportion of recent migrants were significantly associated only in the EU/EEA countries. What these results tell of the implementation of screening programmes or guidelines, is debatable, but it does seem that the higher migration pressure might partially explain differences in practices amongst different countries.

The common higher frequency of screening activities, potentially in relation to migratory pressure, as well as the similar proportion of countries implementing screening programmes targeting newly arrived migrants in the EU/EEA and in non EU countries, support the idea of a conceptual shift from a eurocentric vision of migration with a predominant South-North trend to a more regional “migration system-like” approach [20]. As not all migrants reaching northern Africa or the Balkans manage to reach their final destinations in Europe, some may opt to stay in the “transit country” or move to another within the region. For this reason, all EU neighbouring countries have been associated with transit migration and are becoming increasingly long term or final destinations for a growing number of mixed migrants. In this perspective, migration, and its management, is also a concern for the “transit countries” [21].

To our knowledge this is the first study performed to assess whether migration is considered an issue for ID transmission in EU neighbouring countries of the Mediterranean and Black Sea and to provide an overview of existing ID screening practices targeting newly arrived migrants. In addition, by replicating the methodology used in a similar study among EU/EEA countries [8], we were able to compare the expressed opinions, policies and practices. This comparison also highlighted the relevance of mapping and exchanging existing practices among EU and non-EU Member States.

Consistently with the descriptive and scoping approach of our study, we adopted a very wide definition of screening. The advantage of this choice was that the survey was able to detect different ID screened according to various methods. The disadvantage was that we assessed screening practices on a very broad scale. For this reason, we did not acquire specific information on the screening methods used and other programme details and did not explore differences in screening practices by ID.

As our study describes current existing national screening practices, we did not explore the decision making process that led to the establishment of each programme. Moreover, we did not explore the benefits and harms of screening in the context of current policies nor the screening protocols and laboratory tests adopted in each country.

Our analysis on the proportion of refugees in the population took into consideration only one subgroup of migrants, but did not take into account other migrant groups. In addition, when comparing the results to the earlier EU/EEA survey, the selected migrant groups were different. Therefore, our analysis and comparison only gave a partial view on the situation.

## 5. Conclusions

There is a perceived usefulness and frequent implementation of ID screening programmes targeting newly arrived migrants across the Mediterranean and Black Sea, as well as in the European Union. This might lead to concurrent implementation of similar screening programmes leading to the duplication of testing and vaccination offer targeting the same moving population. Further studies are needed to compare screening practices and derived actions among countries located across the same migration routes, regardless whether they are or not members of the EU/EEA. At the same time more in depth studies are needed to explore in detail the screening methods used for ID screening in the different countries, how each programme is implemented and explore if differences exists in screening practices across countries by ID. Our findings also highlighted that there may be areas for improvement of screening programmes targeting newly arrived migrants in this region and that there may be a need for further guidance on this topic.
